# Immunological abnormalities as potential biomarkers in Chronic Fatigue Syndrome/Myalgic Encephalomyelitis

**DOI:** 10.1186/1479-5876-9-81

**Published:** 2011-05-28

**Authors:** Ekua W Brenu, Mieke L van Driel, Don R Staines, Kevin J Ashton, Sandra B Ramos, James Keane, Nancy G Klimas, Sonya M Marshall-Gradisnik

**Affiliations:** 1 Population Health and Neuroimmunology Unit, Faculty of Health Science and Medicine, Bond University, Robina, Queensland, Australia; 2 Faculty of Health Science and Medicine, Bond University, Robina, Queensland, Australia; 3 Queensland Health, Gold Coast Public Health Unit, Southport, Gold Coast, Queensland, Australia; 4 Miami Veterans Affairs Medical Center, Miami, FL, USA

## Abstract

**Background:**

Chronic Fatigue Syndrome/Myalgic Encephalomyelitis (CFS/ME) is characterised by severe prolonged fatigue, and decreases in cognition and other physiological functions, resulting in severe loss of quality of life, difficult clinical management and high costs to the health care system. To date there is no proven pathomechanism to satisfactorily explain this disorder. Studies have identified abnormalities in immune function but these data are inconsistent. We investigated the profile of markers of immune function (including novel markers) in CFS/ME patients.

**Methods:**

We included 95 CFS/ME patients and 50 healthy controls. All participants were assessed on natural killer (NK) and CD8^+^T cell cytotoxic activities, Th1 and Th2 cytokine profile of CD4^+^T cells, expression of vasoactive intestinal peptide receptor 2 (VPACR2), levels of NK phenotypes (CD56^bright ^and CD56^dim^) and regulatory T cells expressing FoxP3 transcription factor.

**Results:**

Compared to healthy individuals, CFS/ME patients displayed significant increases in IL-10, IFN-γ, TNF-α, CD4^+^CD25^+ ^T cells, FoxP3 and VPACR2 expression. Cytotoxic activity of NK and CD8^+^T cells and NK phenotypes, in particular the CD56^bright ^NK cells were significantly decreased in CFS/ME patients. Additionally granzyme A and granzyme K expression were reduced while expression levels of perforin were significantly increased in the CFS/ME population relative to the control population. These data suggest significant dysregulation of the immune system in CFS/ME patients.

**Conclusions:**

Our study found immunological abnormalities which may serve as biomarkers in CFS/ME patients with potential for an application as a diagnostic tool.

## Background

Chronic Fatigue Syndrome/Myalgic Encephalomyelitis (CFS/ME) remains a medically unexplained disorder despite numerous scientific investigations undertaken worldwide. The current worldwide prevalence rate of CFS/ME is estimated to be about 0.5% [1] with a higher prevalence in females compared to males, at a ratio of up to 6:1 [2]. The annual cost for treatment and management of CFS/ME in the USA is estimated to be US$319 million with a direct cost of US$7,406 per patient [3].

Generally, patients with CFS/ME experience severe fatigue, neuropsychological impairments, and other associated flu-like symptoms before a firm diagnosis of CFS/ME is made [4]. CFS/ME has been observed to persist for more than six months where symptoms may decrease, remain stable or worsen [3]. The current diagnostic strategy for health professionals is based on case definition, although this is not the most ideal method as it permits misdiagnosis. CFS/ME may share homology with certain disorders classified as fatigue related disorders where individuals experience fatigue and one or more of CFS/ME related symptoms. Further, there are no biomarkers available to affirm diagnosis thus complicating treatment.

Population based studies have suggested a link between infections, neurological and neuroimmune dysfunctions and clinical manifestations of CFS/ME [5-10]. Immunity has been widely investigated in patients with CFS/ME but the results of these studies are inconsistent, reporting different lymphocyte cell numbers and cytokine distributions in patients with CFS/ME. Nonetheless, findings on immunoglobulins, complement markers and activation molecules in CFS/ME, may demonstrate an underlying infringement in immune function [8,11,12]. Decreased function of lymphocytes, in particular Natural Killer (NK) cell cytotoxic activity in CFS/ME patients compared to healthy controls, seems to be a consistent finding [13-16]. The functional capacity of other immune cells, such as T cells, and the contribution of other molecules in the pathophysiological mechanism of CFS/ME, remains to be determined. In particular, the role of subsets of CD4^+^T and the CD8^+^T cell populations has not been fully studied in CFS/ME. Importantly, recent data on cytokine distribution in CFS/ME patients point towards an increase in pro-inflammatory cytokines suggesting the presence of an underlying viral prevalence in these patients [17,18] and this can be detrimental to the immune inflammatory processes.

It is widely known that neuropeptides regulate immunity. Relevant among these are vasoactive neuropeptides (VNs), specifically vasoactive intestinal peptide (VIP) and pituitary adenylate cyclase-activating polypeptide (PACAP). They regulate and suppress pro-inflammatory immune processes via the PKA/cAMP pathway [19]. Their role in CFS/ME remains unknown although there are suggestions that they may be implicated in CD4^+^T cell related activities such as cytokine secretion and FoxP3 expression [20].

Immune cell numbers may not necessarily be indicative of diseased states, as stated previously these have been shown to be inconsistent in CFS/ME. However, the functional capacity of these cells during disease progression may provide a better understanding of the mechanism associated with unexplained disorders such as CFS/ME. Alternatively, this may help in identifying specific immune parameters that can be used as diagnostic markers for CFS/ME. The present study thus explores immunological abnormalities that may serve as biomarkers for diagnosing CFS/ME. Additionally, this is the first study to examine the role of VNs, VIP and PACAP, and FoxP3 expression in CFS/ME.

## Methods

The project having been reviewed under an Expedited Review Procedure was granted approval to proceed by the Bond University Human Research Ethics Committee (BUHREC). All participants in this present study signed an informed consent approved by the Bond University Human Research Ethics Committee (BUHREC).

### Participants

**Figure 1:**
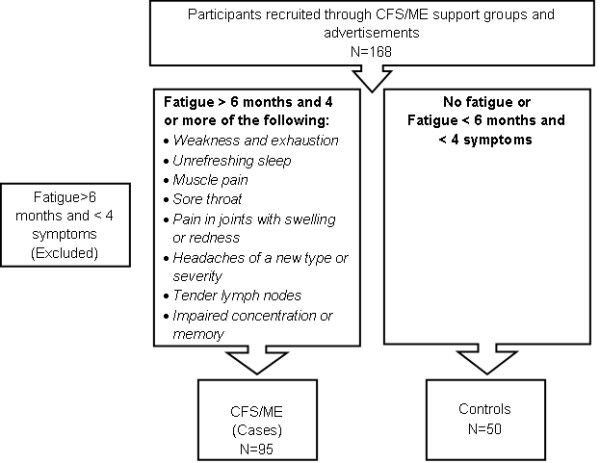
**Selection Process for Experimental Groups**. Participants for the present project were grouped into CFS/ME, or non-fatigued control groups based on the CDC 1994 case definition symptom criteria. Participants, that is, both CFS/ME and non-fatigued controls, were comprised of both male and females selected using advertisements and through the CFS/ME support groups. Non-fatigued controls were randomly selected from the general population using newspaper and email advertisements. The above flow chart illustrates the process used to generate the final research population.

All participants, both CFS/ME and non-fatigued controls were recruited from Queensland and New South Wales states in Australia through the CFS/ME support groups, newspaper and email advertisements into a prospective study as cases (CFS/ME patients) or non-fatigued controls (healthy volunteers). Participants were eligible if they were between 25 and 65 years old. Prior to inclusion all participants completed a consent form and a Chronic Fatigue Syndrome questionnaire based on the Centre for Disease Prevention and Control case definition (CDC 1994) [4]. Participants previously diagnosed with autoimmune disorders, psychosis, epilepsy, heart disease, or who were pregnant or breastfeeding were excluded from the study (Figure 1).

### Sample Preparation and Routine Measurements

A volume of 25 ml of blood was collected from the antecubital vein of participants into lithium heparinised and EDTA collection tubes between 9 am and 11 am. Blood samples were analysed within 12 hours of collection. Routine blood cell counts for red blood cells, lymphocytes, granulocytes and monocytes were performed using an automated cell counter (ACT Differential Analyzer, Beckman Coulter, Miami, FL).

### Assessment of NK Cytotoxic Activity

NK cells were isolated from whole blood samples using Ficoll-Hypaque (GE Healthcare Life Sciences; Milan, Italy) density gradient centrifugation. NK lymphocyte cytotoxic activity was performed as previously described [21]. Briefly, isolated cells were labelled with 0.4% PKH-26 (Sigma, St Louis, MO). NK cells were incubated with K562 cells at an effector to target ratio of 25: 1, for 4 hours at 37°C in 95% air, 5% CO_2_. Apoptosis of the tumour cells was measured via FACS-Calibur flow cytometry using the Cell Quest Software (Becton Dickinson (BD), San Diego, CA), using Annexin V-FITC and 7-AAD reagent (BD Pharmingen, San Diego, CA). Percent lysis of K562 cells was calculated as previously described [21].

### Assessment of CD8^+^T lymphocyte Cytotoxic Activity

Peripheral blood mononuclear cells (PBMCs) were isolated from whole blood samples using Ficoll-Hypaque (GE Healthcare Life Sciences; Milan, Italy) density gradient centrifugation. CD8^+^T lymphocytes were preferentially isolated from PMBCs using CD8^+^T cell isolation kit (Miltenyi Biotec GmbH; Bergisch-Gladbach, Germany) according to the manufacturer's instructions. Briefly, cells were stained with a CD8^+^T cell biotin-antibody cocktail, incubated for 10 minutes and then stained with CD8^+^T cell micorbead cocktail for 15 minutes. Cells were then passed through separation columns where cells of interest were collected for further analysis. Cytolysis was performed as previously described using P815 cells as the target cells [22]. In brief, P815 cells were stained with 0.4% PKH-26 and activated using anti-CD3 (BD Bioscience, San Diego, CA). The target cells were then incubated with CD8^+^T cells at an effector to target ratio of 25: 1, for 4 hours at 37°C in 95% air, 5% CO_2_. Annexin V-FITC flow cytometry apoptosis detection was used in assessing cell death of the tumour cells. Percent lysis of P815 cells was calculated as previously described [22].

### Gene Expression in NK and CD8^+^T cells

Isolation of NK and CD8^+^T cells was done via MACS separation (Miltenyi Biotec GmbH; Bergisch-Gladbach, Germany) as specified by the manufacturer. Purity was determined on the flow cytometer using the Cell Quest software. Isolated NK cells were coated with PE-CD56CD16 and FITC-CD3 (BD Pharningen, San Diego, CA) monoclonal antibodies to determine the purity of NK cells. To establish the purity of CD8^+^T cells, isolated CD8^+^T cells were stained and incubated with PE-CD8 and FITC-CD3 monoclonal antibodies (BD Pharmingen, San Diego, CA). Cells were fast frozen in liquid nitrogen and kept in negative 80 degrees freezing conditions for further assessment. Total RNA extractions were performed using the RNeasy Mini Kit (Qiagen, Valencia, CA) and quantified on the NanoDrop 3300 (Thermo Scientific, Wilmington, DE). RNA was synthesised in to cDNA using the SuperScript™ III First-Strand synthesis SuperMix for qRT-PCR (Invitrogen, Carlsbad, CA) as specified by the manufacturer and stored at negative 20°C for later analysis. RT-qPCR was performed using IQ SYBR Green Super Mix (Bio-Rad, Hercules, CA) with *GAPDH *as the housekeeping gene. Expression levels of granzyme A, granzyme K, perforin and interferon (IFN)-γ (*GZMA, GZMK, PRF1 *and *IFN-*G) genes were collected and quantified using the iQCycler (Bio-Rad, Hercules, CA).

### Quantification of NK Phenotypes

Distribution of NK cell phenotypes was assessed as previously described [23]. NK lymphocytes were isolated from whole blood via negative selection using RosetteSep Human Natural Killer Cell Enrichment Cocktail (StemCell Technologies, Vancouver, BC) and were labelled with CD56-FITC and CD16-PE monoclonal antibodies (BD Pharmingen, San Jose, CA).

### VPACR2 Stimulation

Whole blood samples (10 mL) diluted with 1x PBS were layered over Ficoll-Hypaque for isolation of peripheral blood mononuclear cells. Cells were stimulated with or without 1 μg of Lipopolysaccharide (Invitrogen, Carlsbad, CA) and cultured for 48 hours. Cells were stained with vasoactive intestinal peptide receptor 2 (Sigma, St Louis, MO), FITC-IgG (Sigma, St Louis, MO) and CD4-PE anti-mouse monoclonal antibodies and analysed on the flow cytometer with settings for detecting monocytes and lymphocytes expressing the VPACR2 [24]. Percentage of cells expressing both CD4-PE and VIP2-FITC were recorded from these populations to determine the levels of VPACR2 expressed on these cells. In the lymphocyte gate specific reference was made for CD4^+^T cells.

### Cytokine Determination

Isolated PBMCs were mitogenically stimulated with 1 μg of phytohemagluttinin and cultured at a concentration of 1 × 10^6 ^cells/mL for 72 hours. Following incubation, supernatants were removed and stored at -80°C for later assessment. T helper (Th)1, Th2 and Th17 cytokine expressions were investigated using the cytometric bead array kit (BD Pharmingen, San Diego, CA) [25] for determining levels of interleukin (IL)-2, IL-4, IL-6, IL-10, tumour necrosis factor (TNF)-α, INF-γ and IL-17A. The cytokines selected for this study although not conclusive, enough were selected to ascertain the Th1/Th2/Th17 mechanisms in CFS patients.

### Regulatory T Cell Assessment

Expression of FoxP3 Tregs was determined on CD4^+^CD25^+ ^cells. PBMC Cells were stained with monoclonal antibodies FITC-CD4 and APC-CD25 (BD Pharmingen, San Diego, CA) following which cells were permeablised and stained with anti-FoxP3 and PE-Foxp3 respectively and analysed via flow cytometery [26].

### Statistical Analysis

Statistical analyses were performed using SPSS software version 16.0 (SPSS Inc, Chicago, USA). A sample size of 59 participants per group was required to obtain statistically significant results with an effect size of 0.5 and a power of 85%. All data represented in this study are reported as means plus orminus standard error of the mean (± SEM). Comparative assessments among participants (the CFS/ME and control subjects) were performed using the analysis of variance test (ANOVA) and independent sample t-test. All statistically significant results had *p*-values less than or equal to 0.05.

### Ethical Clearance and Participant Selection

Approval for this study was granted after review by the Bond University Human Research Ethics Committee (RO852A).

## Results

Of the 168 participants recruited 95 met the CDC criteria for CFS/ME and 50 qualified as healthy controls. Twenty-three participants were rejected because they did not meet the inclusion criteria for CFS/ME (Figure 1). 58.2% of CFS/ME patients indicated that they experienced 6 or more of the symptoms listed in the CDC criteria list and 21.4% experienced only 4 symptoms. The baseline characteristics of the participants are illustrated in Table 1.

**Table 1:** Characteristics of participants in the study

Parameters Measured	CFS/ME (n = 95)	Controls (n = 50)	*p*-values
Sex	Female 70.5%	Female 57.7%	
	Male 29.5%	Male 42.3%	
Mean Age	46.47 ± 11.7	41.9 ± 9.6	0.11
Height (cm)	167.47 ± 13.2	167.9 ± 8.9	0.16
Weight (lbs)	169.8 ± 140.7	159.8 ± 46.5	0.11
White Blood Cells	5.8 ± 1.4	6.3 ± 1.8	0.68
Lymphocyte (%)	38 ± 5.7	33.6 ± 7.9	0.03
Monocytes (%)	5.90 ± 1.4	5.6 ± 2.2	0.30
Granulocyte (%)	56.3 ± 7.1	60.8 ± 8.2	0.06
Lymphocyte (x10^3^/μL)	2.3 ± 0.8	2.03 ± 0.6	0.24
Monocytes (x10^3^/μL)	0.8 ± 4.2	0.34 ± 0.2	0.51
Granulocyte (x10^3^/μL)	3.3 ± 1.0	3.87 ± 1.5	0.21
Red Blood Cells (x10^6^/μL)	4.3 ± 0.5	4.56 ± 0.4	0.08
Heamoglobin (g/L)	131.6 ± 12.4	137.0 ± 11.7	0.15
Hematocrit (%)	43.8 ± 3.3	43.68 ± 13.0	0.89

### Lymphocyte Cytotoxic Activity

**Figure 2:**
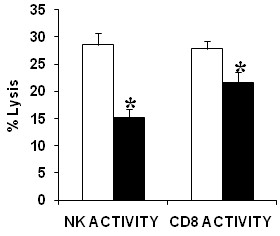
**Reduced lytic function of cytotoxic cells in CFS/ME**. *In vivo *assessment of NK and CD8^+^T cell lysis (cytotoxic activity) of tumour cell lines K562 and P815 respectively in CFS/ME (black bars) in comparison to controls (white bars). Lytic activity is represented as percent (%) lysis on the y-axis. *Denotes statistical significant results. Data presented as mean ± SEM.

**Figure 3:**
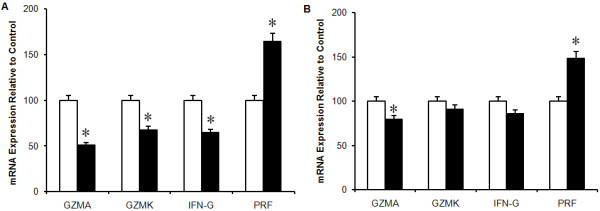
**mRNA Expression of cytotoxic molecules in NK and CD8**^**+**^**T cells**. Quantitative reverse transcriptase (RT)-PCR demonstrated the relative expression of granzyme A, granzyme K, perforin and IFN-γ in NK (**A**) and CD8^+^T cells (**B**). In NK and CD8+T cells expression levels of *GZMA, GZMK *and *IFN-G *were decreased in CFS/ME (black bars) compared to the controls (white bars). *PRF1 *was however increased in CFS group. *Denotes statistical significant results (*P *≤ 0.05). Data presented as mean ± SEM.

NK and CD8^+^T (n = 71) cytotoxic activity measured as the ability of NK and CD8^+^T cells to effectively lyse K562 and P815 cells respectively was significantly decreased (*p *< 0.05) among the CFS/ME patients compared to the control subjects (Figure 2). Similarly granzyme A expression was significantly decreased in both the NK and CD8^+^T cells in the CFS/ME population. However, IFN-γ and granzyme K were decreased only in the NK cells of the CFS/ME group compared to the healthy controls as shown in Figure 3A and 3B.

### Altered NK Profiles in CFS/ME

**Figure 4:**
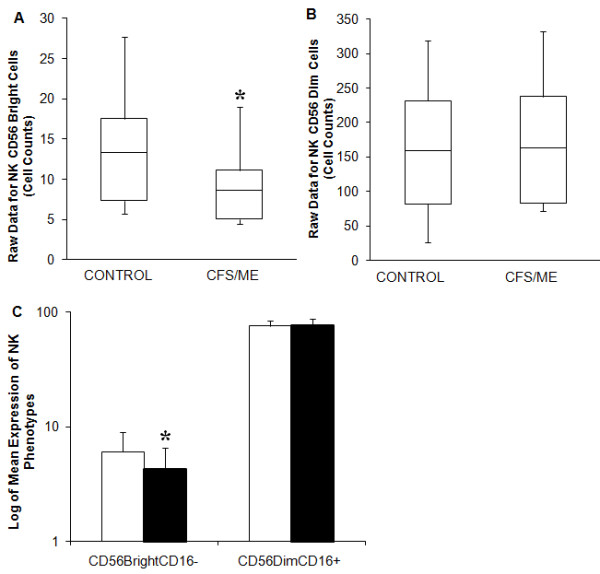
**Distribution of NK phenotypes**. NK phenotypes that were examined are denoted as either NK Bright (CD56^bright^CD16^-^) or NK Dim (CD56^dim^CD16^+^). **(A)**. The box plots represent the raw data of NK Bright cells in the two groups. CFS/ME patients were more decreased in the cell numbers for this particular NK phenotype. **(B)**. However raw data of CD56^dim^CD16^+^NK cells were examined in the control and CFS/ME groups, these were found to be similar. **(C)**. Using the raw data from the flow cytometry results, total counts of NK cells were deduced. These measurements are plotted using bar graphs, CD56^bright^CD16^-^NK cells are more reduced in the CFS/ME (black bars) group in comparison to the controls (white bars). *Denotes statistical significant results (*P *≤ 0.05). Data presented as mean ± SEM.

For the purposes of this study NK phenotypes were classified into two, these are the CD56^bright^CD16^- ^and CD56^dim^CD16^+^NK cells. The number of NK cells expressing CD56^bright^CD16^- ^was significantly lower (*p *< 0.001) in the CFS/ME patients compared to the control subjects (Figure 4C). However, CD56^dim^CD16^+^NK cells remained unchanged across all groups (Figure 4C). The raw data are presented in 4A and 4B.

### Profile of CD4^+^T cells Cytokines and VPACR2 in CFS/ME

**Figure 5:**
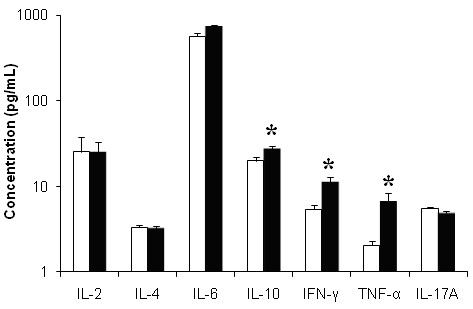
**Examination of the expression levels of CD4+T cell Related Cytokiness in CFS/ME following mitogenic stimulation**. CD4^+^T cells, Th1, Th2 and Th17 cytokine levels in CFS/ME (black bars) and control participants (white bars) measured after mitogenic stimulation with PHA. The concentrations of cytokines were measured in pg/mL. Both anti-inflammatory (IL-10) and pro-inflammatory (IFN-γ, TNF-α) cytokines were increased in the CFS/ME group following mitogenic stimulation. *Statistically significant results at p < 0.05. Data presented as mean ± SEM.

**Figure 6:**
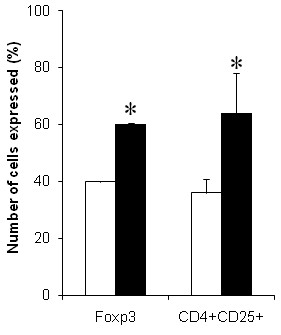
**FoxP3 expression and CD4+CD25+T cells in CFS/ME**. The percentage of CD4+T cells expressing CD4+CD25+FoxP3+ markers are represented in the bar graph. Tregs of interest in this study were those positive for FoxP3 and CD4+CD25+ in CFS/ME (black bars) and control (white bars) participants. *Represent statistically significant results at p < 0.05. Data presented as mean ± SEM.

**Figure 7:**
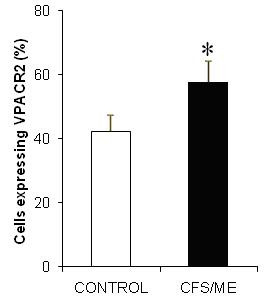
**VPAC2R immune cells in CFS/ME**. VPAC2R expression on CD4^+^T cells was assessed in CFS/ME (black bars) and controls (white bars). The data presented here are based on percentage of cells positive for CD4 and VPACR2. *Represent statistically significant results at p < 0.05. Data presented as mean ± SEM.

After 72 hours of culture Th1 and Th2 cytokine secretions were considerably different between groups, however, Th17 cytokine IL-17A remained unchanged. However, IL-10, IFN-γ and TNF-α production was significantly elevated in the CFS/ME group compared to the control group (Figure 5). Other cytokines IL-2 and IL-6 although increased in the CFS/ME population were not statistically different between groups (Figure 5). IL-17A was similarly not significantly different between the two groups. FoxP3 secretion by Tregs was significantly higher in the CFS/ME group compared to healthy participants (Figure 6). Incidentally, Treg cell counts were also higher in the CFS/ME group compared to the healthy population (0.77 ± 0.10 vs. 0.24 ± 0.02). Lymphocyte expression of VPACR2 was significantly higher in the CFS/ME patients compared to the control group (Figure 7).

## Discussion

This is the first study to show significantly higher levels of VPACR2 receptors, CD4^+^CD25^+^Tregs and FoxP3^+^Treg expression in CFS/ME patients compared to healthy controls. In addition, CFS/ME patients had significantly higher levels of anti-inflammatory cytokine IL-10 and pro-inflammatory cytokines IFN-γ and TNF-α. This profile reflects significant and important immunological dysregulation that could explain some of the clinical symptoms, for example the ongoing sickness experience of CFS/ME patients.

This is the first study to provide a thorough investigation of the CD4^+^T cell profile in CFS/ME patients through the assessment of cytokine secretion and regulatory protein levels in particular VPACR2 receptors and FoxP3 expression. Cytokines are soluble proteins with either anti-inflammatory or pro-inflammatory effects. Equivocal cytokine expression patterns in CFS/ME patients have been reported without a definite identification as to which cytokines may be specifically linked to CFS/ME. Possible explanations for the inconsistencies in cytokine distribution across studies are the heterogeneous nature of the disorder and differences in analytical methods used. However, newer and more sensitive assays have been developed since the conflicting results were reported [17]. It has been suggested that the mechanism underlying CFS/ME may involve a shift in cytokine production leading to either a predominant Th1 or Th2 cytokine profile [27-29]. In the adaptive immune system, CD4^+^T cells subsets, Th1, Th2, Th17 and regulatory T cells (Tregs) are the main regulators of cytokine secretion and the inflammatory immune response. A bimodal Th1/Th2 response was observed in the present study. A predominant Th1 and Th17 immune response has been linked to the development or presence of an autoimmune disease whereas increases in Th2 cytokines suggest the presence of other systemic disorders [30,31]. Th1 cells secrete cytokines IFN-γ and IL-2 while Th2 cells secrete cytokines IL-4 and IL-10 [32] and Th17 secrete pro-inflammatory IL-17a, IL-17f and IL-22 [33,34]. Recent data on cytokine networks in CFS/ME show a predominant Th2/anti-inflammatory profile in CFS/ME with a weakened Th1 profile [17]. This study supports the presence of a possible imbalance in Th1/Th2 response in CFS/ME characterised by a significant increase in IL-10 together with significant increases in IFN-γ and TNF-α. Such increases in IL-10 are suggestive of a persistent chronic infectious state and may be associated with a dampening of the NK and CD8^+^T cell immune response [22]. Others have shown that IL-10RA is differentially expressed in CFS/ME patients, highlighting a potential compromise in IL-10 function or its receptor in CFS/ME patients [35,36]. Nonetheless, increased levels of IL-10, IFN-γ and TNF-α indicate the presence of fungal, bacterial or viral infection [37]. Incidentally in HIV elevation in IL-10, IFN-γ and TNF-α denote the presence of a chronic infection and this correlated with viral load [38]. Similarly in CFS such alterations in these cytokines may also suggest an increase in viral load and the occurrence of flu-like symptoms. An increase in IL-10 also may contribute to decreased cytotoxic activity observed in the NK and CD8^+^T cells [39,40]. The increase in pro-inflammatory cytokines such as TNF-α, may also depict the presence of an inflamed gut or irritable bowel syndrome in some CFS/ME patients [41]. Inflammation in the gut can alter the central nervous system [42,43] and affects various physiological mechanisms including neuropeptides.

The changes in both the Th1 and Th2 responses may suggest changes in the function of VN receptor VPACR2 which is a key promoter and stimulator of anti-inflammatory cytokines such as IL-10 [44]. It is important to note that VNs, VIP and PACAP have never been assessed in CFS/ME previously. These important neuropeptides increase IL-10 gene expression via the cAMP response element DNA binding complex pathway, therefore changes in VNs such as elevations in VPACR2 may suggest an increase in IL-10 [45]. Further, an increase in TNF-α and IFN-γ suggests an inability of the heightened VPACR2 to suppress TNF-α and IFN-γ secretion as these neuropeptides are noted to suppress pro-inflammatory cytokines while favouring anti-inflammatory cytokine secretions [46]. Additionally, increases in VPACR2 potentially suggest changes in cAMP associated with the inflammatory immune response in CFS/ME. Although our study did not assess the levels of cAMP present in CFS/ME patients, VIP binding to its receptor, in this case VPACR2, is known to stimulate the presence of FoxP3^+ ^which assists in regulating the T cell response. Thus it is consistent that heightened levels of VPACR2 will translate into heightened FoxP3 expression. FoxP3^+ ^Tregs also secrete IL-10 which maintains the expression of FoxP3 in Tregs [47]. The increased expression of IL-10 and the relatively higher expression of FoxP3 together with significant increases in CD4^+^CD25^+^Tregs suppressive activity suggest a requirement to counter a significant pro-inflammatory response in these patients. While levels of viral antigens were not measured in this study, these observations may suggest a plausible prevalence in viral antigens, adjuvants or autoantibodies in the peripheral circulation of CFS/ME patients [48,49].

NK cytotoxic activity in CFS/ME has received much attention while only one study has examined CD8^+^T cell cytotoxic activity. Most studies found significant decreases in NK activity and one study found decreased CD8^+^T cell cytotoxic activity in a CFS/ME population compared with a control group. These findings are confirmed in our study. In a previous study [13] as well as this study in a larger population we have found that NK cytotoxic activity and CD56^bright ^NK phenotypes are decreased in CFS/ME patients. These atypical cytotoxic responses may be linked to compromised granule-mediated cell death pathways involving apoptotic mediators, perforin and granzymes. Perforin forms pore-like structures to facilitate the entry of granzymes into the target cell [50], and granzymes activate several apoptosis pathways that ensure effective killing of the target cell [51]. Perforin and granzymes have been shown to be decreased in both NK and CD8^+^T cells in CFS/ME [16,52]. In contrast both granzyme A and granzyme K were significantly reduced while perforin levels were elevated in both the NK and CD8^+^T cells of CFS/ME patients. Reduced cytotoxic activity may therefore be an important component of the immune dysregulation seen in CFS/ME.

## Conclusions

These results illustrate a severely compromised immunomodulation mechanism in CFS/ME where attempts to regulate or restore immune homeostasis appear to be impaired. These findings suggest that certain immunological biomarkers as demonstrated in this study may be unique to CFS/ME. To date no routinely available clinical immunological markers have been identified that characterise CFS/ME, resulting in poor recognition and management of patients. The immunological abnormalities identified in our study can potentially fill this void as potential biomarkers and assist clinicians and patients in diagnosis and management of this severely debilitating condition. These biomarkers may include NK phenotypes, NK activity, CD8^+^T cell activity, IL-10, IFN-γ TNFα, FoxP3 and VPACR2. These markers that seem to be unique to CFS/ME patients could assist in identifying them as a distinct population, enabling more appropriate clinical management and better targeted scientific investigations into the underlying pathomechanisms of the disease.

## Authors' contributions

Conceived and designed the experiments: EWB DRS SMMG. Performed the experiments: EWB SBR JK. Analysed the data: EWB. Supplied reagents/analysis tools: SMMG MVD KJA. Wrote the paper: EWB DRS SMMG MVD. Critically reviewed paper: SMMG, KJA, MVD, DRS, NGK. All authors read and approved the complete manuscript.

## Competing interests

The authors declare that they have no competing interests.
